# Growth and development of the ovary and small follicle pool from mid fetal life to pre-puberty in the African elephant *(Loxodonta africana)*

**DOI:** 10.1186/1746-6148-8-119

**Published:** 2012-07-23

**Authors:** Fiona J Stansfield, Johan O Nöthling, William R Allen

**Affiliations:** 1 Department of Production Animal Studies, Faculty of Veterinary Science, University of Pretoria, Onderstepoort, Republic of South Africa; 2 The Elephant Research and Conservation Unit, Savé Valley Conservancy, Chiredzi, Zimbabwe; 3 The Paul Mellon Laboratory, “Brunswick”, 18 Woodditton Road, Newmarket, Suffolk, CB89BJ, UK

**Keywords:** Elephant, Oogenesis, Folliculogenesis, Interstitial cell, Ovary, Reproductive life, Follicle pool

## Abstract

**Background:**

Follicle numbers and developing ovarian morphology, particularly with reference to the presence of interstitial tissue, are intimately linked within the ovary of the African elephant during the period spanning mid-gestation to puberty. These have not been previously quantified in any studies. The collection of 7 sets of elephant fetal ovaries between 11.2 and 20.2 months of gestation, and 29 pairs of prepubertal calf ovaries between 2 months and 9 years of age during routine management off-takes of complete family groups in private conservancies in Zimbabwe provided an opportunity for a detailed study of this period.

**Results:**

The changing morphology of the ovary is described as the presumptive cortex and medulla components of the fetal ovary settled into their adult form. Interstitial tissue dominated the ovary in late fetal life and these cells stained strongly for 3β–hydroxysteroid dehydrogenase. This staining continued postnatally through to 4.5 years of age suggesting continued secretion of progestagens by the ovary during this period. The considerable growth of antral follicles peaked at 28% of ovarian volume at around 16.7 months of fetal age. The numbers of small follicles (primordial, early primary and true primary), counted in the cortex using stereological protocols, revealed fewer small follicles in the ovaries of animals aged 0 to 4.5 years of age than during either late fetal life or prepubertal life.

**Conclusions:**

The small follicle populations of the late-fetal and prepubertal ovaries of the African elephant were described along with the changing morphology of these organs. The changes noted represent a series of events that have been recorded only in the elephant and the giraffe species to date. The expansion of the interstitial tissue of the fetal ovary and its continued presence in early post natal life may well contribute to the control of follicle development in these early years. Further research is required to determine the reasons behind the variation of numbers of small follicles in the ovaries of prepubertal calves.

## Background

The number of follicles in the ovarian reserve of mammals is established during fetal life or early in neonatal life [1]. From this reserve a number of follicles are recruited each day, the majority of which become atretic while a very few develop to the point of ovulation in each oestrous cycle [2]. The biological norm is therefore to show a steady loss of small follicles throughout life with the resulting depletion of the follicle reserve [3]. This dogma has been challenged in recent years and the debate on neo-oogenesis and the existence of post-natal oogonial stem cells (OSC) which can transform into meiotic, fertile oocytes continues [4,5].

The follicle reserve in the African elephant has been found to be composed of early primary follicles (ePF; oocytes surrounded by a single layer of pregranulosa cells, some of which may be cuboidal in shape) and true primary follicles (tPF; oocytes surrounded by a single layer of pregranulosa cells, all of which are cuboidal). True primordial follicles (PrF); an oocyte surrounded by a single layer of pregranulosa cells, all of which are flat and none cuboidal) form <2% of the follicle reserve [6]. The oocytes are larger in tPF than in ePF follicles [6], suggesting that follicle activation [7] may have taken place and that tPF do not belong to the ovarian reserve but form part of the SF pool in the African elephant.

Female elephants, along with whales, show an extended reproductive lifespan which, apart from the absence of menopause, is remarkably similar to that of women; namely, puberty at age 10–12 years, full cyclicity and fertility to age 50 and a slow decline in fertility thereafter followed by senescence and death at age 65–70 years [8-10]. It is therefore of interest to study reproductive strategies and mechanisms in female elephants and, in particular, to examine the reserves of follicles in their ovaries throughout life.

Considerable growth and enlargement of the fetal gonads occurs during the second half of gestation in the elephant [11,12] and numerous antral follicles grow and subsequently undergo atresia during this period [13].

Interstitial cells are found in the fetal gonads of many mammalian species including the elephant [14]; human [15], spotted hyena [16], guinea pig [17], rock hyrax [18] and in some—for example the horse [19] and the Common and Grey seals [20]—they cause a significant increase in fetal gonadal volume. In the female fetus in most of these species, the interstitial cells develop independent of follicles within the presumptive ovarian medulla. In the elephant [12] and the giraffe [21,22] however, the interstitial cell hyperplasia and hypertrophy not only takes place independently but also occurs in association with antral follicle development during the second half of gestation. In the elephant, where these cells have been studied in fetuses up to the age of approximately 21 months, have been shown to be steroidogenically active, with the potential to synthesise progestagens from cholestorol and pregnenelone [14]. Their fate after birth is unknown.

The present study aimed to describe the development of the elephant ovary and its pools of small follicles and medullary interstitial cells during the second half of fetal life and the first nine years of postnatal life.

## Results

### Fetal ovarian morphology (11–20 months of gestation)

**Figure 1:**
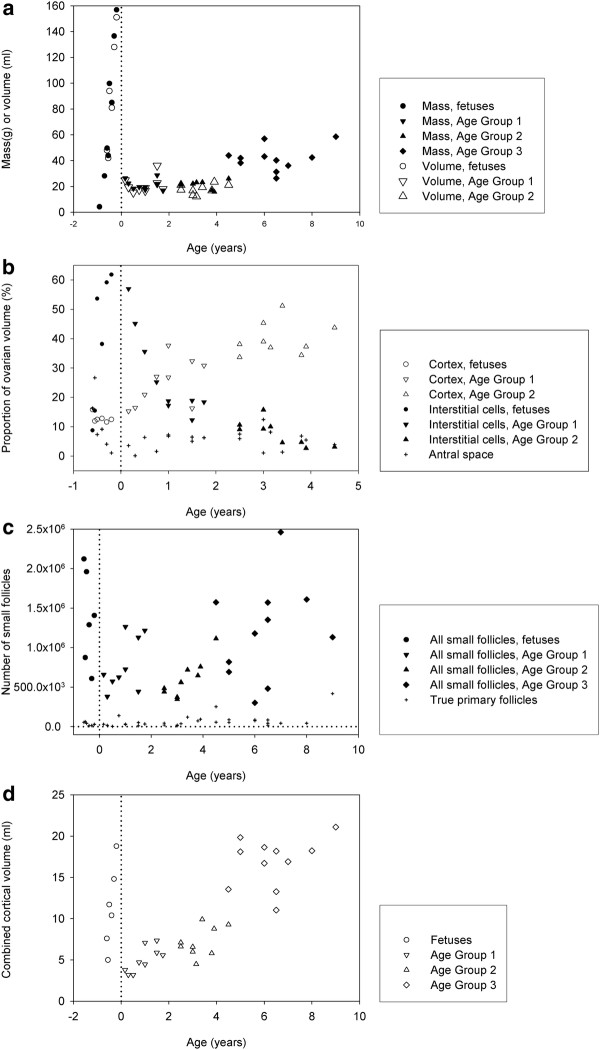
** (a) Combined weights and volumes of both ovaries from African elephant fetuses from mid-gestation onwards and calves up to 9 years of age (volumes were calculated using Cavalieri’s principle).** (**b**). Relative contributions of cortex, interstistial cells and follicular antra to the volumes of the ovaries from African elephant fetuses (from mid-gestation onwards) and calves up to 4.5 years of age (volumes were calculated using Cavalieri’s principle. (**c**). The number of small follicles in the elephant fetus and prepubertal calf in relation to age. (**d**). Cortical volume of both ovaries combined of fetal and prepubertal ovaries of African elephants (volumes were calculated using Cavalieri’s principle.

**Figure 2:**
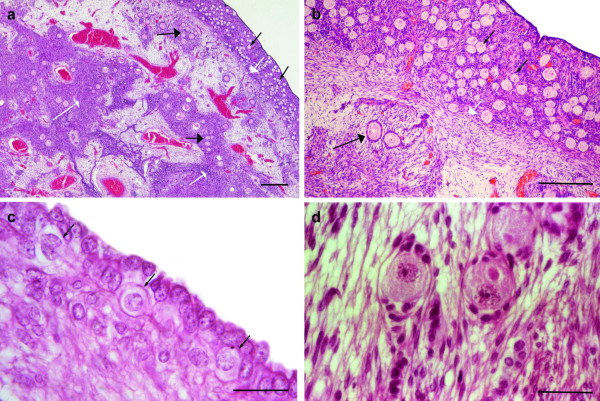
** Sectioned ovary of an elephant fetus at 11 months of gestation.a**) Section from the surface epithelium to the central medulla showing small follicles throughout the cortex (small black arrows). The cortico-medullary border is indicated by a white arrow. In the medulla black arrows mark groups of pre-antral follicles while the smaller headed white arrows show areas of interstitial tissue (Scale bar = 500 μm). **b**) A higher magnification of the cortex and cortico-medullary border (indicated by the white arrow). Small follicles within the cortex are indicated by small black arrows and growing follicles within the medulla by the larger black arrow (Scale bar = 250 μm. **c**) The surface epithelium of the ovary consisted of a monolayer of cuboidal cells and in places, juxtaposed to these epithelial cells were cells with the size and appearance of oogonia (black arrows; Scale bar = 20 μm). **c**) Early primary follicles showing the typical prolate shape (Scale bar = 40 μm).

The combined mass and volumes of the ovaries of each elephant fetus and calf are shown in Figure 1a. A description of the ovaries from the 11.2 month old fetus is given below in order to describe the basic morphology of the gonad before the enlargement associated with the second half of gestation has commenced. In this fetus at mid-gestation the left ovary weighed 0.25 g and the right 0.27 g. The cortex and medulla were well defined (Figure 2a), the cortex being approximately 450 μm in depth and composed of remnants of ovigerous cords, small follicles and cortical stroma consisting of fibrocytes and mesenchymal cells (Figure 2b). The ovarian surface epithelium consisted of a monolayer of cuboidal cells with occasional oogonia visible juxtaposed to these epithelial cells (Figure 2c). In some sections the *tunica albuginea* formed a narrow layer of constituent cells running parallel to and just beneath the ovarian surface. Follicles present in the cortex were predominantly early primary in development (Figure 2d) and measured 50.0–65.0 μm, (oocyte 45–55.0 μm, nucleus 20–22.5 μm*).* A vascular network was clearly visible deep in the cortex. The presumptive medulla was strongly demarcated by the presence of dark staining interstitial cells which had no particular association with follicles (Figure 2a). Large blood vessels and *rete ovarii* were also observed within the medulla. Growing small follicles up to the stage of small antral follicles with a diameter of 1.6 x 1.0 mm were present within the medulla but these were spread more sparsely than follicles in the cortex (Figure 2b).

**Figure 3:**
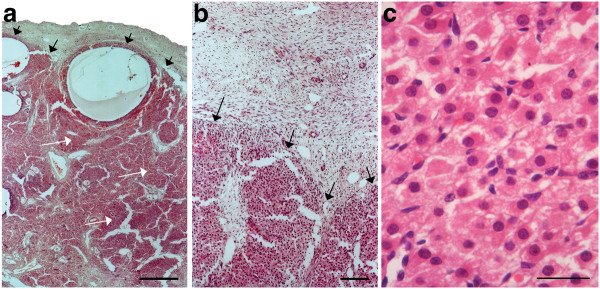
** Sections of the ovaries of an elephant fetus at 13.5 months of gestation.a**) The black arrows indicate the cortico-medullary border. The white arrows indicate interstitial tissue (Scale bar = 700 μm). **b**) The border between cortical tissue at the top of the photograph and interstitial tissue at the bottom (Scale bar = 350 μm). **c**) High power magnification of the interstitial cells shown in b (Scale bar = 20 μm).

**Figure 4:**
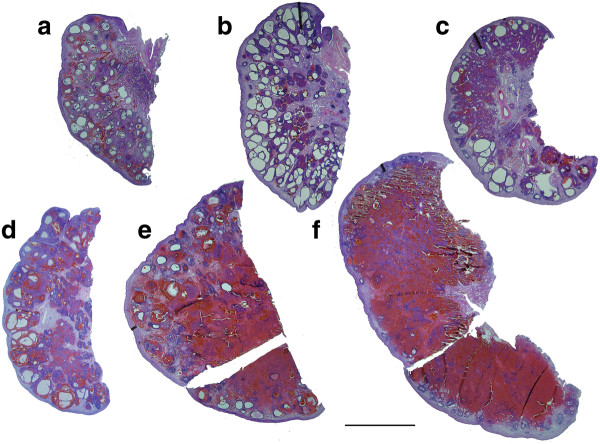
** Photographs taken above a light box of 25 μm thick sections of fetal elephant ovaries recovered between 15 and 21 months of gestation (Scale bar = 10 mm).a**) 15.2 months; the cortical region surrounds a central medulla filled with small growing and antral follicles and dark staining interstitial tissue. **b**) 16.7 months; showing maximum recorded antral follicle development. **c**) 17.1 months; the number of antral follicles is less than in (b) and more interstitial tissue is apparent. **d**) 17.5 months; increasing vascularisation of the ovary is apparent. **e**) 19.3 months; ovarian size has increased significantly from (d) due to hypertrophy and hyperplasia of interstitial cells. **f**) 20.2 months; the fetal ovary is approaching maximum size. Few antral follicles remain and, as in e), the interstitial tissue is partially obscured by extravasated red blood cells.

In the fetus at 13.5 months of gestation the left ovary weighed 14.0 g and the right 14.2 g and antral follicles were present within the medulla and along the cortico-medullary border (Figure 3a). The medulla appeared more highly vascularised and now contained prominent accumulations of darkly staining interstitial cells (Figure 3b). These were polyhedral in shape with dark staining nuclei and eosinophylic cytoplasm (Figure 3c) and were appreciably larger than those of the granulosa cells or other stromal cells. The medullary interstitial cells appeared to be continuous with the *theca interna* cells of growing and atretic antral follicles which now reached diameters of 1–2 mm. Within this presumptive medulla were patches of undifferentiated light-staining adipose-rich mesenchymal cells. The cortical region of the fetus at 15.2 months (Figure 4a) contained oocytes that typically showed a prolate association of granulosa cells similar to those shown in Figure 2d in early primary follicles. These formed a narrow band beneath the surface epithelium. Early primary follicles measured 37.5–46.0 μm, the oocytes 25.0–31.0 μm and their nuclei 15.5–17.5 μm. The left ovary weighed 27.5 g and the right 21.5 g.

Maximum follicle growth within the specimens studied was observed around 16.7 months (Figure 4b) when antral follicles of 3–5 mm diameter were present in the medulla to give a total antral volume of around 11.2 ml in both ovaries combined. The well vascularised cortex had a depth of 400–500 μm and the ovarian surface remained smooth. The combined cortical volume of the two ovaries of the 20-month fetus was 18.8 ml, which was about 2.5 times larger than that of the 15-month fetus (7.6 ml), although the proportional contribution to ovarian volume was the same (Figure 1b) because the ovarian volume of the 20-month fetus was much larger than that of the 15-month fetus.

At 17 months of gestation (Figure 4c and 4d) distinctly fewer antral follicles were visible in the central medulla where highly vascularised groups of interstitial cells now predominated throughout the stroma of the medulla and these contributed up to 54% of the volume of the ovary (Figure 1b). Patches of dense interstitial cells or theca cells marked atretic antral follicles and glassy remnants of basal membranes could also be seen. The cortex was typically 375–875 μm deep.

At 19 months of gestation distinctly fewer and smaller antral follicles were observed in the medulla compared to ovaries from younger individuals, with those present being confined to the cortical border, the cortex being typically 375–1125 μm in width. The bundles of interstitial cells appeared less densely crowded together than in previous samples (Figure 4e).

**Figure 5:**
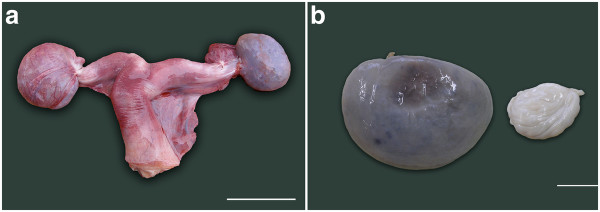
** a) The uterus and ovaries of an elephant fetus at 17.5 month of gestation.** The ovary on the left is still enclosed within its ovarial sac (Scale bar = 50 mm). b) Ovary from a 20.2 month old elephant fetus on the left and a 6 month old calf on the right showing great shrinkage around the time of birth due to the reduction in both the size and number of interstitial cells (Scale bar = 20 mm).

Blood oozed from the cut surface of each purple coloured ovary from fetuses observed at 19 and 20.2 months of gestation, illustrating the intense vascularization of particularly the medulla at these later stages (Figure 5a and 5b). Many fewer antral follicles were present and these were smaller than in the ovaries of younger fetuses, and they were now distributed only along the medullary-cortical border (Figure 4f). Interstitial cell bundles were widely spread throughout the medulla and in the fetus at 20.2 months occupied approximately 93.3 ml which was about 60% (Figure 1b) of the total volume (151 ml) of the two ovaries (Figure 1a).

### Prepubertal calf ovarian morphology

**Figure 6:**
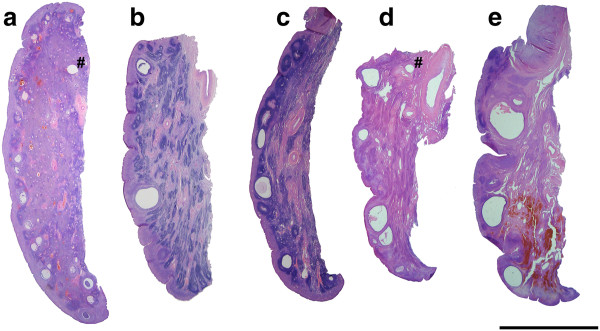
** Photographs of 25 μm thick sections through the ovaries of prepubertal African elephant calves (Scale bar = 10 mm).a**) At 2 months of age, abundant interstitial tissue is visible within the medulla. Several small antral follicles are seen on the cortico-medullary border, these are now regarded as being in the cortex even though they may bulge into the medulla. Artifacts marked with a # are pin holes. **b**) At 1 year of age, the cortical depth is greater than in (a) and isolated larger antral follicles may be seen. Interstitial tissue is present in streaks within the medulla. **c**) At 1.5 years of age, interstitial tissue is visible within the medulla and antral follicles are observed more frequently than in (b). **d**) By 3 years of age, the ovary is settling into its adult pattern of antral follicles residing wholly within the true cortex. **e**) At 4.5 years, the ovary is now starting to enlarge due to a considerable increase in the volume of the cortex.

In the youngest female calf, examined at 2 months of age, ovarian weight (13.1 and 13.4 g) and volume were dramatically lower than those of late fetal life (Figure 1a), predominantly because of a significant reduction in the volume of interstitial cells and the organisation of extravasated blood (Figure 5b). The cortical tissue appeared to have a higher density of cells per unit volume than in fetal life, fewer blood vessels were observed and the cortical depth ranged from 450 to 1000 μm (Figure 6a). The distinctly fewer and smaller (2 x 2 mm) antral follicles along the cortico-medullary border occupied <2% of the ovarian volume. By two months after birth the islands of interstitial tissue which, with blood vessels and remnants of atretic follicles, occurred throughout the medulla were smaller than those observed during late fetal life and occupied only 14 ml per animal compared to 93 ml in a 20-month old fetus. Furthermore, more stromal cells were observed among the interstitial cells than was the case in younger animals. At 4.5 years of age interstitial tissue had reduced to 3% of ovarian volume (Figure 1b). Cortical depth varied from 2 to 3 mm and became further extended by the presence of antral follicles.

During the early months after birth scattered deposits of haemosiderin were observed throughout the ovary reflecting the previously mentioned intense vascularisation of the tissue in late fetal life. The distribution of tissues within the postnatal ovary is detailed in Figure 1b. By 6 years of age interstitial tissue occupied <1% of ovarian volume and the general morphology of the ovary had settled into its “adult” form (Figure 6e).

### Immunohistochemical staining of interstitial cells

**Figure 7:**
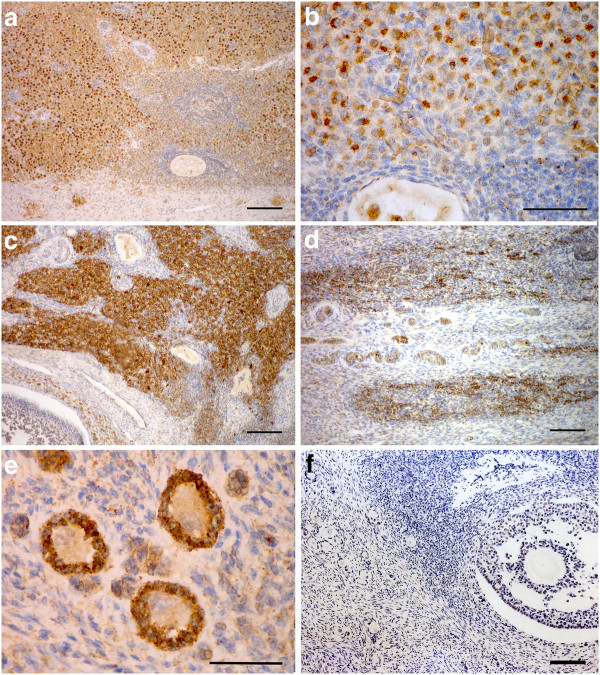
** Sections of the ovaries of elephant fetuses and calves stained immunocytochemically with an anti-3β–HSD antibody.a**) Interstitial cells in a fetal ovary at 17.5 months of gestation (Scale bar = 350 μm). **b**) Higher magnification of the interstitial tissue showing the intense, granular staining for 3β–HSD activity (Scale bar = 40 μm. **c**) Positively stained interstitial tissue in the ovary of a 2 month old calf (Scale bar = 350 μm). **d**) 3β–HSD staining of groups of interstitial cells in the ovary of a 2.5 year old calf (Scale bar = 350 μm). **e**) The granulosa cells of small follicles within the cortex (and medulla in the fetal ovary) stained precisely and strongly for 3β–HSD activity in all the samples examined between 16 months of gestation and 5 years of age (Scale bar = 15 μm). **f**) Negative control showing the complete ablation of staining following replacement of the primary anti-3βHSD antibody with an unrelated mouse monoclonal antibody (Scale bar = 350 μm).

The whorls of interstitial cells in the fetal ovary stained with increasing intensity (Figure 7a) during the second half of gestation for 3β-hydroxysteroid dehydrogenase (3β–HSD), the steroidogenic enzyme necessary to convert pregnenelone to progesterone and other progestagens, as described previously by Allen *et al.*[14]. 

Initially, from around 11 months of gestation the cytoplasm of the 3β–HSD positive cells stained evenly and diffusely but, again as gestation advanced, groups of interstitial cells were observed in which the staining pattern within the cytoplasm became very intense and granular (Figure 7b). The thecal cells of the growing preantral and antral follicles (from about 400 μm in diameter) also stained positively but less intensely. Of great interest was the finding that the interstitial cells continued to stain strongly for 3β–HSD activity after birth (Figure 7c). The incidence and intensity of staining declined steadily during the first 2 years of postnatal life although there were still small patches of 3β–HSD positive interstitial cells visible at 2.5 years of age (Figure 7d). Of interest also was the 3β–HSD positive staining of the cytoplasm of the granulosa cells surrounding the small follicles (Figure 7e) which began around 16 months of gestation. Prior to this only the oocyte cytoplasm had stained lightly. This cyctoplasmic staining of the granulosa cells continued in the SF present in the cortex, and those in the medulla, during fetal life and up to at least 5 years after birth. With progression to the secondary stage of follicle development and onward, the granulosa cells no longer stained positively.

#### Ovarian weight

Figure 4 demonstrates the change in ovarian size with age. Ovarian mass differed by age group (P < 0.001), such that the ovaries of fetuses at 15–20 months of gestation were appreciably larger than those of calves between 2 months and 4.5 years in age.

#### Follicle number

Figure 1c shows the numbers of small follicles per elephant for fetuses and calves of different ages. No primordial follicles (small follicles with a single layer of granulosa cells of which all are flat) were seen. Early primary follicles (small follicles with a single layer of granulosa cells, of which most are flat and few cuboidal) were abundant and constituted between 63 and 99% of all small follicles. True primary follicles (small follicles with a single layer of cuboidal granulosa cells) were less abundant; the maximum observed was 419 717 per individual animal (Figure 1c). The number of early primary follicles as well as the total number of small follicles depended upon age (P = 0.01). Table 1 shows that the numbers of early primary follicles and small follicles were higher in fetuses than in calves aged 2.5–4.5 years. Table 1 further shows that the numbers of early primary follicles and small follicles were higher in calves aged 4.5–9 years than in the younger calves, aged 2.5–4.5 years. The number of true primary follicles was lower in calves aged 0.16–2 years than in calves aged 4.5 years or older (Table 1). Cortical volume increased with age in prepubertal calves (Figure 1d).

**Table 1:** The median (95% confidence interval) combined number of small follicles in both ovarian cortices of African elephant fetuses and prepubertal calves

**Age group**	**Age (years)**	**n**	**Type of small follicle**		
			**Early primary**	**True primary**	**All**
0	-0.6 to –0.16^a^	6	1336570 (596440–1957260)^b^	37506 (13390–62875)	1348101 (609623–2123103)^b^
1	0.16–2	9	627680 (433039–1181875)	31298 (12827–52498)^b^	658978 (445867–1216548)
2	2.5–4.5	9	523065 (359989–664542)^cd^	45268 (19857–95113)	561023 (373157–759654)^cd^
3	4.5–9	11	1093978 (463416–1530106)^e^	76118 (43085–88172)^c^	1180948 (482007–1575054)^e^

^a^ Ages below zero refer to fetuses and indicate the fractions of a year before birth.

^b,c,d,e^ Within a column, medians marked with ^b^ differ from those marked with ^c^ and those marked with ^d^ differ from those marked with ^e^ (P < 0.05).

## Discussion

### Late fetal and prepubertal ovarian morphology

The foregoing described the morphological development and differentiation of the ovaries of the African elephant and the numbers of small follicles they contain, from 11 months of gestation to a prepubertal age of 9 years. Although each measurement and description of the ovaries were independent observations made in different individuals, the pattern of change over time is suggestive of development and in the absence of longitudinal studies provides the only currently available data.

At 11 months gestation very few naked oogonia remain identifiable within the cortex of the fetal ovary and nearly all the germ cells are present as meiotic oocytes arrested at the dictyate stage of prophase I [23] and surrounded by a single layer of granulosa cells which vary in shape from flat to cuboidal. These follicles within the cortex appear to form the follicle reserve for future reproductive life. The many follicles observed within the medulla of the elephant fetal ovary do not share this future and are destined for growth, beginning around 12 months gestation, to small antral stages before the onset of atresia which may occur at any developmental stage thereafter up to mid antral size. Fetal ovarian weight and volume increase from 11 months with the onset of interstitial cell hyperplasia and the growth of medullary antral follicles. By 15 months, as described previously by Allen *et al.* (2005), the increase is significant. The maximum number of antral follicles is observed around 16 months following which they decline to occupy <2% of ovarian volume at birth. Meanwhile, the volume of interstitial tissue continues to grow toward a maximum point thought to occur just prior to birth (22 months), although this could not be confirmed in the present study as the oldest set of fetal ovaries collected was at 20.2 months of gestation. Loss of small follicles and pre-antral follicles has been observed in other mammalian species during fetal life [24]. The reasons for this loss are speculated upon [25,26] and are now generally referred to as natural wastage. The present findings suggest, however, that the elephant fetus may employ its medullary follicles for a useful purpose; ie the production of greater amounts of interstitial tissue as discussed below. In turn, the rising steroid production within the fetal ovary may halt the further development of antral follicles.

How and why the small follicles within the cortex are protected from recruitment to pre-antral growth and the fate of atresia is not known, nor is it known in other species what causes activation of some primordial follicles and not others during fetal and post natal life [27]. There is, however, evidence that either primordial follicles [28] or the local environment [29] may be involved. What seems likely from this elephant study is that recruitment is intimately related to local environment, this being stimulative in the medulla and restrictive in the cortex during the second half of fetal life.

During postnatal life a remarkable feature of the elephant ovary is the persistence of interstitial tissue within the medulla, otherwise it develops in a manner similar to the bovine [30] and other mammals.

### Interstitial cells

Interstitial cells within the mammalian ovary are not static components [31]. They differentiate from stromal fibroblasts, either independently (primary) or when they become associated with growing follicles [2,32] and they revert to this cell type, either independently or following follicle atresia [2].

In equids, interstitial cells in both the fetal testis and fetal ovary begin to multiply rapidly in the absence of any follicle or seminiferous tubule growth from around day 80 of gestation. This interstitial cell hyperplasia and hypertrophy continues unabated to days 220–250 of gestation when the fetal ovaries weighing 50–100 g each are considerably heavier than the now-inactive maternal ovaries [19,33]. The tightly packed epithelioid interstitial cells secrete large quantities of 19-carbon androgen precursors, including androstenedione and dehydroepiandrosterone (DHEA), [34,35] and the very unusual 3-ß hydroxy-5,7 pregnanedien-20-one and 3-ß hydroxy-5,7 androstadien-17-one [36] which are then aromatised by the placenta to produce the relatively enormous (μg/ml) quantities of both phenolic (oestrone and oestradiol) and Ring B unsaturated (equilin and equilenin) oestrogens that are present in the blood and urine of pregnant mares during the second half of gestation [37,38] and which appear to be important for growth and development of the very precocious equine fetus at birth [39]. Similar interstitial tissue development results in ovarian hypertrophy in the Grey and Common seals, also without antral follicle formation [20]. In both horses and seals the interstitial tissue declines rapidly in late gestation so that the gonads have shrunk to their normal prepubertal size at birth [19,20,40].

As described by Hanks [41] and Allen *et al.*[12], and confirmed in the present study, gonadal enlargement also occurs in the elephant fetus during the second half of gestation due to a similar hyperplasia and hypertrophy of primary interstitial cells augmented by secondary interstitial tissue that persists following atresia of antral follicles. And as also demonstrated in the present study, these hypertrophied primary interstitial cells stain for the steroidogenic enzyme, 3-β hydroxysteroid dehydrogenase (3β–HSD) which indicates that they are capable of synthesising progestagens. Indeed, Allen *et al.*[14] demonstrated that slices of elephant fetal gonad incubated with tritium-labelled cholesterol or pregnenelone secreted appreciable quantitites of 5-∂ dihydroprogesterone (5-∂ DHP) and other 5-∂ pregnane derivatives into the culture medium. More recently, Yamamoto *et al.*[42] demonstrated the secretion of placental lactogen (elPL) by elephant trophoblast tissue and speculated that this chorionic hormone may be the essential luteotrophic stimulus for the enlargement of the fetal gonads and their synthesis of progestagens to assist the accessory corpora lutea in the maternal ovaries to maintain the pregnancy state. The highly vascularised nature of the fetal ovary from 18 months of gestation to term would help to transport the progestagens being synthesized by the interstitial tissue to the fetal, and hence to the maternal circulation to boost the supply of progestagens for pregnancy maintenance [43].

An unexpected and interesting finding in the present study was the persistence of 3β–HSD positive nests of interstitial cells in the ovaries of female elephant calves after birth. Such steroid-secreting tissue accounted for some (30–40%) of the total volume of the ovary during the first 6 months of life and it declined slowly thereafter to disappear completely only at around 4.5 years of age. It seems reasonable to speculate that continued secretion of progestagens by these interstitial cells during early post natal life may act locally to suppress any significant growth of antral follicles during the period, and indeed, very little antral follicle development occurs in the first 1–2 years of life in the elephant calf. A few antral follicles develop later in the second year of life, coincidentally with the disappearance of the interstitial tissue and the rate of antral follicle growth increases markedly from 4.0 to 4.5 years onwards when the whole ovary begins to increase in size. Further research is planned to explore the steroid output of these post-natal cells.

Also of interest was the 3β–HSD positive staining of the granulosa cells of small follicles from 16 months of gestation till the oldest studied sample at 5 years of age (Figure 7e) as indeed do the Sertoli cells of the testis at the same ages (F J Stansfield and W R Allen unpublished data). Light staining for 3β–HSD has also been described in pig [44], sheep [45] and human [46] fetal ovaries. It is highly irregular to obtain such a precise and specific staining for 3β–HSD in the granulosa cells of SF during what is considered to be a gonadotrophin independent stage of follicle development [47]). In the current study, the granulosa cells of follicles beyond the secondary stage of development did not stain positively. It is normal in mammalian ovaries for the production of androgens to take place exclusively in the theca cells of the developing follicle [48] and theca cells are usually first observed when the follicle has >2 rows of granulosa cells. In the rat these theca cells are capable of producing steroids just prior to antrum formation [49,50]. In this study 3β–HSD stained theca cells of small antral follicles starting around 400 μm in diameter were observed from 16 months of gestation to 5 years of age.

### Follicle numbers

The stereological measurement of the numbers of small follicles in the ovaries of 29 prepubertal elephant calves and 6 late gestation fetuses revealed a wide between-animal variation in numbers (range 303 084–2 456 741) during the period; the close agreement in follicle numbers between the two ovaries of each animal gave great confidence as to the validity of the counting method [51]. Natural variation in small follicle numbers between animals of similar age is commonly observed in many mammalian species. For example, Schmidt *et al.*[52] revealed variations of more than two orders of magnitude following cortical biopsies of human ovaries and Hansen [53] showed similar results in a review of small follicle numbers in 122 women from birth to 51 years of age.

The number of small follicles fell during late fetal life, as observed in many mammalian species [48]. These follicles were evenly distributed between the left and right ovaries as observed previously [51] and follicle density decreased coincidentally with the age-associated increase in cortical volume of the ovary (Figure 1d). No true primordial follicles were observed so, as in the previous studies [6,54], the small follicle pool was taken as being composed basically of early primary and true primary follicles.

The unexpected finding that the numbers of early primary follicles and the numbers of small follicles in calves aged 4.5–9 years was higher than those in younger calves raises a number of interesting questions as to how such an unusual situation might occur. Firstly, it should be acknowledged that, given the small number of calves included in the study and the large variation in follicle numbers among individuals, the difference, although statistically significant, may have been due to a Type 1 statistical error, brought about by coincidentally using a group of younger calves with lower numbers of follicles and (or) coincidentally using a group of older calves with higher numbers of follicles. Other possible explanations include genetic diversity and post-natal oogenesis. The additional possibility of error in calculating volume was also examined closely.

Relatively few animals could be included in the study (n = 6 fetuses and 29 calves) and biological variation may indeed have accounted for the apparent increase in numbers of follicles counted after birth [55,56]. The selection of the family group to be shot was based on their presence in the culling area on the day, there was no prior knowledge of the group and all members of the group were killed. The chosen culling area was based on high elephant numbers and was deemed to be a random selection of the female family groups within SVC. It is also noteworthy that nearly all of the original 670 elephant which constituted the founding population of the SVC were introduced during 1991 and 1992 from a closed population in nearby Gonarezhou National Park and no further translocations have been made during the ensuing 20 years while population size has increased by natural breeding alone to an estimated 1 500 animals. Predominantly family groups were introduced originally to the Conservancy so the population is biased towards females with few mature mating bulls. Knowing that in women the age of menopause, brought on by exhaustion of the follicle reserve is highly heritable [53], the degree of genetic diversity of the elephant population sampled could be less than that of a truly wild population.

With regard to the experimental protocol, the calculation of the volume of the cortex may be suspected as a source of error to produce the increasing small follicle numbers. In mammals, the division between the cortex and the medulla of the ovary is fluid [57] and the line of differentiation is set halfway between the occurrence of follicles in the cortex and the blood vessels in the medulla [55]. The volume calculations for the cortex and other compartments within the ovary in the present study of fetal and calf ovaries to 4.5 years of age were made using Cavalieri’s Principle as discussed by Gundersen [58] and have been described previously [51]. These calculations were made on the same slides from which the follicle densities were obtained.

The division between the medulla and cortex is particularly blurred during fetal life. In the youngest fetal ovaries in which follicle counts were made in the present study (15.2 months gestation) the cortex formed a very narrow and distinct band just under the surface epithelium of the ovary, which then contained only small follicles. The medulla was composed predominantly of interstitial tissue and growing small follicles and antral follicles; all follicles would normally be included in the cortical region of the ovary of a calf or adult. The percentage volume of the cortical region containing the small follicles was similar in the fetal and calf ovaries (Figure 1b) despite the very considerable fall in ovarian weight and volume during the neonatal period. This suggests strongly that the cortical volume measurements were accurate in both developmental stages. Post-natally, it is generally accepted [59] that there is no further development of new antral follicles in the medulla and all subsequent follicles are derived from the cortical reserve.

The third possibility of some form of post natal oogenesis occurring in the elephant is particularly intriguing. As mentioned in the Introduction, the localization of oogonial stem cells (OSC) of adult mouse and human ovaries [4,60] and the birth of pups following transplantation of female germ-line stem cells to the ovaries of irradiated mice [61] has raised significant, although still controversial, doubts about the finality of meiotic arrest in the ovaries of all mammals during fetal life. It remains possible that OSC may persist in, or migrate to, the epithelium covering the outermost surface of the cortex of the elephant ovary during early post natal life which could, due to some hitherto unknown stimulus, multiply mitotically within the cortex before entering meiotic arrest as in fetal life prior to acquiring an outer layer of persisting granulosa cells to form new SF and so boost the reserve of these structures.

## Conclusions

The small follicle populations of the late-fetal and prepubertal ovaries of the African elephant were described along with the changing morphology of these organs. The changes noted represent a series of events that have been recorded only in the elephant and the giraffe species to date [62]. The expansion of the interstitial tissue of the fetal ovary and its continued presence in early post natal life may well contribute to the control of follicle development in these early years. Further research is required to determine the reasons behind the 3β–HSD staining of the granulosa cells of small follicles of the specimens in this study, and also the variation of numbers of small follicles in the ovaries of prepubertal calves.

## Methods

### Tissue recovery

During routine management off-takes of complete family groups in the Savé Valley Conservancy, Zimbabwe from 2009 until 2011, 29 prepubertal elephant were shot at close range with heavy calibre rifles. The ovaries were recovered within 1 h after death and marked to denote left and right placement within the body cavity and their lateral versus medial surfaces. In addition, 6 sets of ovaries were collected from the fetuses of mature cows that were recorded as being in the second half of gestation [63]. A 7th set of ovaries from a fetus aged at 11.2 months was collected for morphological and immunohistochemical studies only and a block sample from a 13.5 month fetus collected during a previous study [12] was also referred to. Each ovary was photographed and partially bisected lengthwise before being immersed in approximately 10 volumes of 4% buffered formalin which was replaced completely after one hour. The lower jaw of each elephant was boiled to remove soft tissue, photographed and later used to estimate the age of the animal [8,64]. In pregnant females gestation stage was calculated from fetal weights using the formula devised by Craig [63].

### Tissue preparation

In the laboratory the whole fixed ovaries were weighed and fully bisected. If both ovaries were available from the same animal each half of each ovary was cut into 10 equal segments of about 3–5 mm thickness running perpendicular to the ovarian surface and from the mesovarial ligament to the free margin. For ovaries >5 cm wide, 10 equally spaced segments were cut from each half ovary and processed for histological examination. The segments were identified for ovary (left or right), side of the ovary (lateral or medial), interpolar position (indicating progression from the cranial toward the caudal pole on each surface) and marginal position (identifying the position of observation relative to the mesovarial or free margin). The 5 even-numbered segments taken from each side of each ovary were used for analysis. Stansfield *et al.*[51] showed that the number of small follicles in both ovaries of an elephant can be estimated with an average error of 10.5% from the number counted in one ovary. Where only one ovary was available per animal then 10 segments were used from each side of the one ovary. Following embedding in wax the segments were sectioned at 25 μm thickness and stained lightly with haematoxylin and eosin (H&E). The stained sections were then mounted under coverslips, using DPX mounting medium (Leica, Germany), and used to count small follicles.

### Immunohistological methodology

Normal 5 μm sections cut from 3 widely spaced segments of the fetal and prepubertal ovaries were stained with H&E to study the morphology of the tissues and immunocytochemically to study the steroidogenic potential of both the interstitial cells and the granulosa and *theca interna* cells surrounding primary to antral follicles. For this purpose the paraffin embedded sections were placed in a 56°C oven overnight to remove the wax before they were placed in a pre-heated (68°C) bath of high pH antigen unmasking solution. (Dako Ltd, Ely, Cams, UK) and heated to 97°C for 20 minutes. After cooling the slides were rinsed in neutral buffer and transferred to a Dako Autostainer in which a computer controlled indirect staining method was performed using a mouse monoclonal 3ß-hydroxysteroid dehydrogenase (3ß-HSD) primary antibody (3ß-HSD[2]:SC-100466; Santa Cruz, Biotechnology Inc. Ca USA). A biotinylated goat anti-mouse serum (BA – 9200; Vector Laboratories, Burliname, California, USA) diluted 1:200 was used as a second antibody. Incubations with both the primary and secondary antibodies lasted 30 minutes and blocking reagents, buffer, substrate, chromogen and nuclear stains were all as supplied by Dako Ltd. After staining the slides were removed from the machine, dehydrated, cleaned and mounted in DPX.

### Histological and stereological examinations

Follicles were observed using a BX41 microscope (Olympus) and classified according to Oktay [65] as primordial (single layer of granulosa cells, all flat in shape), early primary (single layer of granulosa cells, some flat and some cuboidal in shape) and true primary (single layer of granulosa cells, all cuboidal in shape). Follicle number estimations were performed with the aid of a digital microcator (Sylvac, Switzerland) and the Optical Brick stereology tool as previously described [51].

Elephant ovaries are relatively large compared to those of other mammalian species studied to date using stereology [53,66,67] and the present protocol was tailored to take account of the low density of small preantral follicles within the ovaries of elephant calves [51]. The stereology was carried out in two steps, i) calculation of the volume of the cortical area of the ovary using Cavalieri’s Principle and, ii) calculation of the number of follicles per unit volume (ml) of the ovary using the optical brick method as described by Howard and Reed [68]. The data did not meet the requirements for parametric tests, and the Kruskal-Wallis test was used to compare medians, with α set at 0.05. The Kruskal-Wallis multiple comparison Z-value test with Bonferroni's adjustment was used for pairwise comparisons among groups, with α set at 0.05. NCSS Statistical Software 2004 (NCSS, Kaysville, UT, USA) was used for statistical analysis.

#### Classification of prepubertal calves into 3 physiological groups

Prior to counting small follicle numbers the cohort of 29 calves was divided into 3 groups based on macroscopic observation of the ovarian sections; Group 1 (about 2 months to 2 years of age, n = 9) exhibited a large volume of interstitial tissue persisting in the ovaries; Group 2 (about 2.5–4.5 years of age, n = 9) showed negligible amounts of interstitial tissue and Group 3 (about 4.5–8 years, n = 11) had markedly larger ovaries than those placed in the other two groups. There were two animals of about 4.5 years, one of which fell into Group 2 and the other into Group 3.

#### Ageing

The animals were aged using Laws’ molar progression [8], the accuracy of which was confirmed recently by observations of molar progression in jaws from elephant of known age dying of natural causes in Amboselli National Park, Kenya [64]. Fetuses in the second half of gestation were aged on the basis of fetal weight using the formula devised by Craig [63].

### Ethical approval

The elephant were culled in terms of permits issued by the Zimbabwean Parks and Wildlife Management Authority. The animals were culled for reasons other than obtaining research specimens and the ovaries in the current study were collected opportunistically.

## Abbreviations

ePF, Early primary follicles; tPF, True primary follicle; PrF, True primordial follicle; OSC, Oogonial stem cell.

## Competing interests

The authors declare there is no conflict of interest that could be perceived as prejudicing the impartiality of the research reported in this paper.

## Authors’ contributions

FJS performed the study design, specimen collection, histological study, stereology study and drafted the manuscript. JON performed the study design, statistical analyses, produced the graph figures and edited the manuscript. TWA assisted in specimen collection, arranged histological processing and immunohistochemical staining, discussed the drafting of, and edited the manuscript. All authors read and approved the final manuscript.
